# Ex vivo drug sensitivity testing as a means for drug repurposing in esophageal adenocarcinoma

**DOI:** 10.1371/journal.pone.0203173

**Published:** 2018-09-13

**Authors:** Ines Lohse, Hassan Al-Ali, Claude-Henry Volmar, Annamil D. Alvarez Trotta, Shaun P. Brothers, Anthony J. Capobianco, Claes Wahlestedt

**Affiliations:** 1 Center for Therapeutic Innovation, Miller School of Medicine, University of Miami, Miami, Florida, United States of America; 2 Department of Psychiatry and Behavioral Sciences, Miller School of Medicine, University of Miami, Miami, Florida, United States of America; 3 Molecular Therapeutics Shared Resource, Sylvester Comprehensive Cancer Center, University of Miami, Miami, Florida, United States of America; 4 Sylvester Comprehensive Cancer Center, University of Miami, Miami, Florida, United States of America; 5 Miami Project to Cure Paralysis, Miller School of Medicine, University of Miami, Miami, Florida, United States of America; 6 Department of Neurological Surgery, Miller School of Medicine, University of Miami, Miami, Florida, United States of America; 7 Peggy and Harold Katz Drug Discovery Center, Department of Medicine, Miller School of Medicine, University of Miami, Miami, Florida, United States of America; 8 Molecular Oncology Program, DeWitt Daughtry Family Department of Surgery, Miller School of Medicine, University of Miami, Miami, Florida, United States of America; 9 Division of Surgical Oncology, DeWitt Daughtry Family Department of Surgery, Miller School of Medicine, University of Miami, Miami, Florida, United States of America; University of Navarra, SPAIN

## Abstract

**Background:**

Esophageal cancer remains one of the hardest cancers to treat with rising incidence rates, low overall survival and high levels of treatment resistance. The lack of clinically available biomarkers hinder diagnosis and treatment stratification. While large scale sequencing approaches have uncovered a number of molecular makers, little has translated in the routine treatment of esophageal cancer patients.

**Material and methods:**

We evaluate the treatment response towards a panel of 215 FDA-approved and 163 epigenetic compounds of 4 established and 2 patient-derived esophageal cancer cell lines. Cell viability was evaluated after 72h of treatment using cell titer glow. The drug sensitivity testing results for gemcitabine and cisplatin were validated using clonogenic assays.

**Results:**

The tested cell lines display different drug sensitivity profiles, although we found compounds that display efficacy in all of the tested established or patient-derived cell lines. Clonogenic assays confirmed the validity of the drug sensitivity testing results. Using the epigenetic library, we observed high sensitivity towards a number of epigenetic modifiers.

**Discussion:**

*Ex vivo* drug sensitivity testing may present a viable option for the treatment stratification of esophageal cancer patients and holds the potential to greatly improve patient outcome while reducing treatment toxicity.

## Introduction

Esophageal cancer is the 8^th^ most common cause of cancer death in Western countries with rising incidence rates of adenocarcinoma of the lower esophagus and esophagogastric junction [1].

Although the prognosis for early stage disease is favorable, overall survival remains low at 19% despite advances in surgical techniques and multimodal therapies [1, 2]. Apart from the ERBB2/HER2 status, no biomarkers that complement or improve the diagnosis, risk stratification and therapy of the disease as part of routine clinical practice [3].

A number of recent high-throughput investigations revealed histological, genetic and epigenetic changes typical for esophageal adenocarcinoma [3]. While, these studies have uncovered a number of mutations and revealed an aberrant methylation of the genome [4–7], little has translated into clinical practice to date. However, a number of clinical trials currently evaluate whether the utility of genomic and epigenetic markers for early detection, prognosis and prediction of response to treatment [4–6, 8]. Optimal pre-clinical staging based on the American Joint Committee on Cancer (AJCC) Staging System based on tumor-node-metastasis (TNM) sub-classifications [2, 9, 10] remains crucial in optimizing outcomes for patients with esophageal adenocarcinoma [2].

Precision medicine approaches for the treatment stratification of patients with esophageal adenocarcinoma have recently come into the focus of the field in order to improve patient outcome. Apart from improvements in PET imaging techniques [11, 12], the lack of available material from biopsies, long turn over times of sequencing approaches and failure to detect targetable mutations have prevented the clinical application [13].

We have overcome these issues by adapting a drug sensitivity testing (DST) platform previously established for the stratification of patients with relapsed/refractory AML [14]. Ex vivo DST screening allows the identification of patient-specific treatments from a library of 215 FDA-approved compounds that are available on compassionate care and may present a new stratification approach for esophageal adenocarcinoma patients [14].

## Material and methods

### Esophageal cell lines

OE33, OE19 and SKGT2 human esophageal adenocarcinoma cell lines were obtained from the European Collection of Cell Culture (ATCC). Flo1, EAC42 and EAC47 cells were established in the Capobianco Laboratory. EAC42 and EAC47 human esophageal adenocarcinoma cell lines are derived from patients undergoing surgery at the Miller School of Medicine, University of Miami [15]. The Capobianco laboratory obtained written informed consent from all patients and approval from the Institutional Research Ethics Committee.

The normal esophageal epithelium cell line EACN42 was established in the Capobianco laboratory. The cell lines was derived from human normal epithelium and immortalized using HPV E6-E7.

### FDA library drug sensitivity testing

Drug sensitivity testing (DST) was performed as described previously [14]. Briefly, a range of 215 FDA/EMA (Food and Drug Administration/European Medicines Agency)-approved anti-cancer drugs were represented in the compound library, covering a variety of targets and pathways relevant to cancer in general and esophageal cancer specifically (Table 1). Individual drugs were dissolved in 100% DMSO and tested in duplicates starting at a maximal test concentration of 10μM then over a 20,000-fold concentration range to generate dose response curves. Wells with assay buffer containing DMSO served as negative controls. 1000 exponentially growing cells were seeded per well in 384-well micro-titer plates and incubated in the presence of compounds in a humidified environment at 37°C and 5% CO2. After 72 hours of treatment, cell viability was assessed by measuring ATP levels via bioluminescence (CellTiter-Glo, Promega) according to manufactures recommendations. Interpretation of curve parameters was performed according to the modified drug sensitivity scoring (DSS_mod_) function developed by Swords et al [14]. As a final step, the selective DSS_mod_ (sDSS_mod_) for each drug in each patient screen is calculated according to the formula sDSS_mod_ = DSS_mod_ (patient cells)—DSS_mod_ (normal cells). Given in this way, the sDSS_mod_ incorporated information on each drug’s potency, efficacy, effect range and therapeutic index, making it possible to prioritize compounds over multiple parameters using a single numerical metric. In addition, this methodology allows us to rank compounds by cancer-selective efficacy for each individual patient. For example, a large positive sDSS_mod_ means that a compound is highly selective for esophageal cancer cells over normal cells in a given sample (favorable scenario), while a large negative score means that the effect was preferential for normal cells (unfavorable scenario). All calculations and scoring routines are implemented in MatLab and additional curve fitting and statistical analyses are performed in GraphPad prism.

**Table 1:** Compound library for *ex vivo* drug sensitivity screening. All listed agents are FDA-approved and classified according to mechanism of action where available.

Class	Compound
Alkylating agents	Bendamustine, Busulfan, Carboplatin, Cisplatin, Cyclophosphamide, Dacarbazine, lfosfamide, lomustine, Methazolastone, Oxaliplatin, Procarbazine, Streptozotocin
Antimetabolites	Azacitidine, Azaguanine-8, Capecitabine, Carmofur, Cladrabine, Clofarabine, Cytarabine, Decitabine, Febuxostat, Floxuridine, Fludarabine, Fluorouracil, Ftorafur, Gemcitabine, lonidamine, Mercaptopurine, Methotrexate, Nelarabine, Pemetrexed, Thioguanine
Antimitotics	10-Deacetylbaccatin, Cephalomannine, Docetaxel, Paclitaxel, Vinblastine, Vincristine
Antitumor antibiotics	Artemether, Azithromycin, Bacitracin, Bleomycin, Hygromycin B, Lincomycin, Methacycline, Ofloxacin
HDAC inhibitors	Belinostat, Panobinostat, Sodium Butyrate, Vorinostat
Hormone Inhibitors	2-Methoxyestradiol, Abiraterone, Aminoglutethimide, Anastrozole, Bicalutamide, Clomifene Citrate, Diethylstilbestrol, Doxercalciferol, Enzalutamide, Exemestane, Flutamide, Fulvestrant, ltraconazole, Letrozole, Megestrol, Mifepristone, Paeoniflorin, Raloxifene, Tamoxifen, Toremifene, Triamcinolone
lmmunomodulators	Aspirin, Azathioprine, Bindarit, Cortisone, Celecoxib, Dexamethasone, Hydrocortisone, lmiquimod, Maraviroc, Meprednisone, Mizoribine, Mycophenolate, Phenylbutazone, Pimecrolimus, Pomalidomide, Prednisone, Sulindac, Tacrolimus, Thalidomide, Vinpocetine, Zileuton
Kinase inhibitors	Afatinib, Apatinib, Axitinib, Bosutinib, Cabozantinib, Crizotinib, Dasatinib, Erlotinib, lbrutinib, lmatinib, Lapatinib, Masitinib, Nilotinib, Pazopanib, Ponatinib, Regorafenib, Ruxolitinib, Sorafenib, Sunitinib, Tofacitinib, Vandetanib, Vemurafenib
Proteasome inhibitors	Bortezomib, Carfilzomib, Ubenimex
Rapalogs	Everolimus, Sirolimus, Temsirolimus
Topoisomerase 1/2 inhibitors	Camptothecin, Daunorubicin, Epirubicin, Etoposide, ldarubicin, lrinotecan, Mitoxantrone, Teniposide, Topotecan
Miscellaneous antineoplastics	Altretamine, Anagrelide, Bexarotene, Eltrombopag, Geniposide, Hydroxyurea, Mitotane, MLN4924, lsotretinoin, Tretinoin
Other	Adenine, Aprepitant, Atazanavir, Bepotastine, Bergapten, Blonanserin, Carbazochrome, Clorsulon, DAPT (GSI-IX), Disulfram, Dorzolamide, Ellagic acid, Epinephrine bitartrate, Esomeprazole, Ezetimibe, Flunarizine, Fluvastatin, Gadodiamide, Genistein, L-Arginine, Lamotrigine, Leucovorin, Linagliptin, Mesna, Mirabegron, Naloxone, Noscapine, Pamidronate, Pioglitazone, Ranolazine, Rosiglitazone, Orthovanadate, Temocapril, Tolbutamide, Valproic acid, Zoledronic acid, Vismodegib

### Clonogenic assay

Clonogenic assays were performed as described previously [16]. Briefly, the patient-derived cell lines EAC42 and EAC47 were treated with 100nM gemcitabine and 50μM or 10 μM cisplatin for 2h and 24h respectively. Colony formation was evaluated 14 days after seeding. All experiments were performed in triplicates and two biological replicates. Statistical analyses were performed in GraphPad prism.

### Epigenetic compound library screen

We tested a 163 compound epigenetic library that consists of inhibitors and activators of epigenetic modifying enzymes (writers, erasers and readers). All compounds were dissolved in 100% DMSO and tested in duplicate at a nominal test concentration of 6μM. Wells with assay buffer containing DMSO served as negative controls. 1000 exponentially growing cells were seeded per well in 384-well micro-titer plates and incubated in the presence of compounds in a humidified environment at 37°C and 5% CO2. After 72 hours of treatment, cell viability was assessed by measuring ATP levels via bioluminescence (CellTiter-Glo, Promega). Positive hits were defined as any compound that showed cell killing higher that 3 standard deviations of the negative control. Curve fitting and statistical analyses are performed in GraphPad prism.

## Results

### Drug sensitivity testing of normal esophageal cells

**Fig 1:**
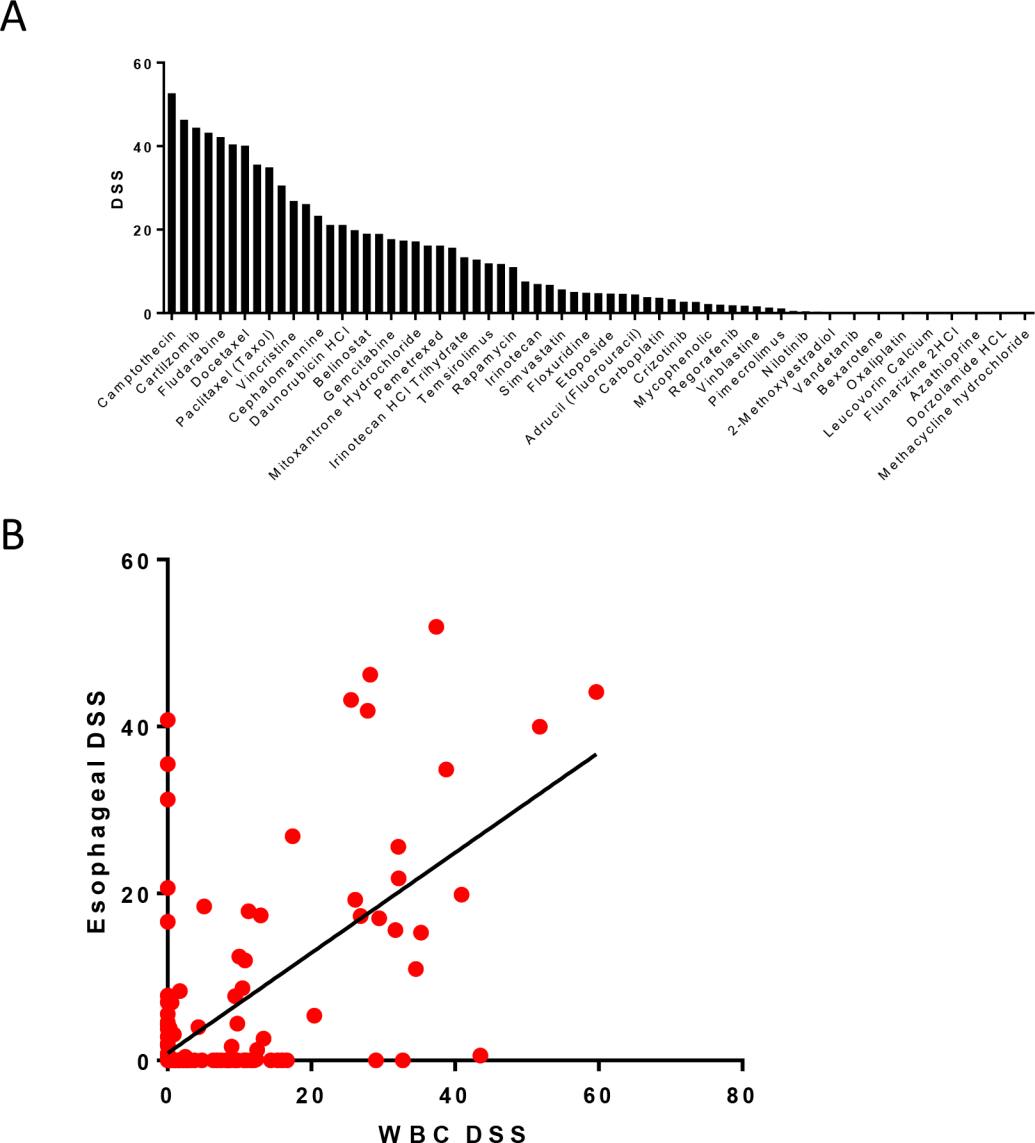
Drug sensitivity profile of the normal control cell line. (A) Bar graphs of treatment responses of the normal esophageal epithelium cell line EACN42. (B) Comparison of response towards the FDA-approved compound library of patient-derived white blood cells and normal esophageal epithelium.

The normal esophageal epithelium cell line EACN42 was exposed to the 215 compound FDA-approved library cell viability was evaluated after 72 of treatment. The esophageal epithelial cells display toxicity to 71 compounds (Fig 1A). Significant responses (DSS ≥5), however, were only observed in response to treatment with 34 compounds. The highest toxicity was observed in cells treated with topoisomerase 1/2 inhibitors (Camptothecin (DSS 52.69), Idarubicin (DSS 26.1), Topotecan (DSS 43.21)) and antimitotics (Docetaxel (DSS 40.14), Paclitaxel (DSS 34.94), Vincristine (DSS 26.09), Cephalomannine (DSS 23.35)) and antimetabolites (Flurada (DSS 46.34), Fludarabine (DSS 42.19)). Immunomodulators (Aspirin, Maraviroc) the other hand display low or no activity (S1 Table). Similarly, alkylating agents such as Cisplatin, Carboplatin and Oxaliplatin only displayed minimal treatment effects.

Because matched normal tissue is not available at sufficient quantities from patients with esophageal adenocarcinoma from either biopsies or surgical intervention, we compared the response of the esophageal epithelium to the treatment response of white blood cells derived from a healthy donor (Fig 1B). While we do observe a correlation in treatment response in a small number of compounds including rapamycin, gemcitabine, capcecitabine and vinblastine, the majority of compounds display vastly different treatment responses.

### Drug sensitivity testing of esophageal cancer cell lines

Drug sensitivity of 4 established and 2 low passage patient-derived esophageal cancer cell lines towards a panel of 215 FDA-approved compounds was established using the DST platform.

**Fig 2:**
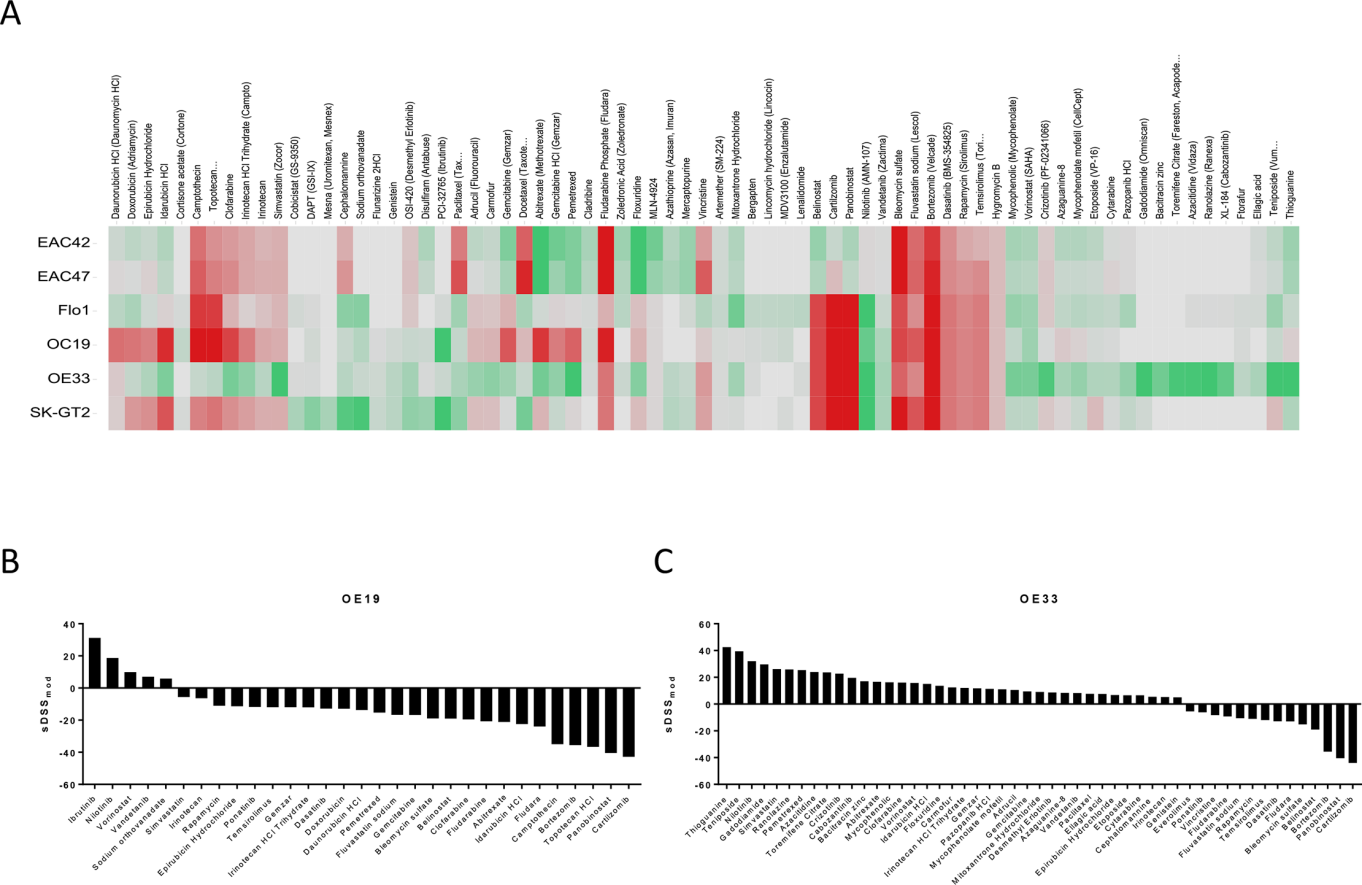
Ex vivo drug sensitivity testing. (A) The heatmap of sDSS_mod_ profiles reveals large variability in both direction and magnitude of drug responses in the esophageal adenocarcinoma cell lines tested. The sDSS_mod_ profile for each cell line is depicted with all drugs that had a score of more than +5 or less than -5 in at least one cell line (drugs that had no effect in any cell linewere excluded). Cell lines and drugs were clustered using hierarchical clustering with a tanimoto distance metric. Red color indicates a positive sDSS_mod_ score while green color indicates a negative sDSS_mod_ score. Bar graphs of clinically actionable drug responses for (B) OE19 and (C) OE33.

Similar to what we have previously observed in AML [14], the 4 tested established esophageal cancer cell lines (OE19, OE33, Flo-1, SK-GT2) display vastly different drug sensitivity profiles (Fig 2, S1 Fig). Nevertheless, we identified a small subset of compounds that displayed activity in all of the cell lines (Belinostat, Carfilzomib, Panbinostat, Bleomycin, Bortezomib, Dasatinib, Rapamycin (Sirolimus) and Temsirolimus) although the magnitude of response was different between cell lines (Fig 2A, S2 Table). The HDAC inhibitor Panbinostat and the proteasome inhibitors Bortezomib and Carfilzomib display the highest treatment efficacy in all cell lines.

**Fig 3:**
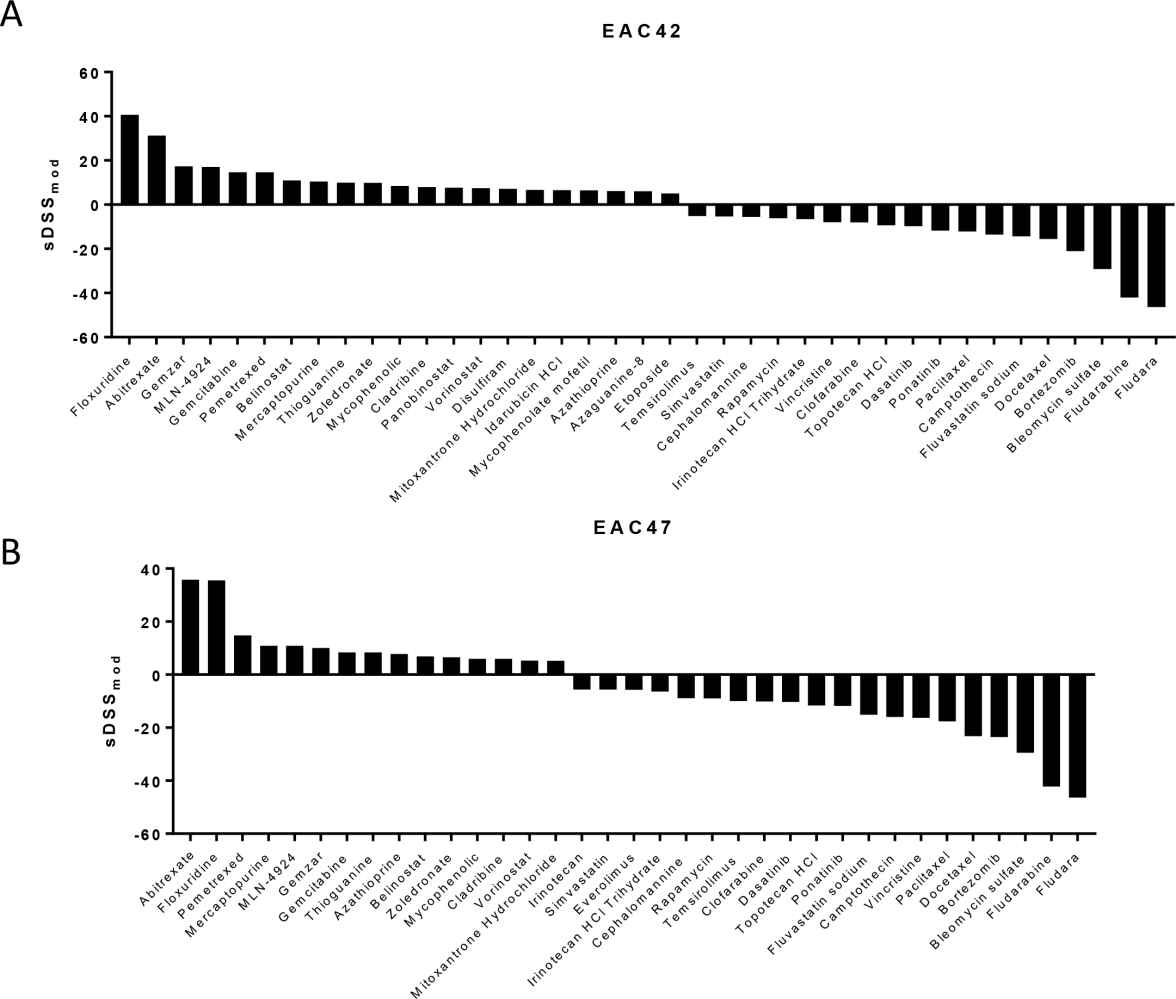
Individual screening results. Bar graphs of clinically actionable drug responses for (A) EAC42 and (B) EAC47.

In addition to the established esophageal cancer cell lines, we evaluated EAC42 and EAC47, two patient-derived esophageal cell lines at low passages. Although both lines differ in the magnitude of response, the drug sensitivity profiles are very similar (Figs 2A and 3). The antimethabolites Floxuridine and Methotrexate (Abitrexate) display the highest levels of cancer selective efficacy (S2 Table).

**Fig 4:**
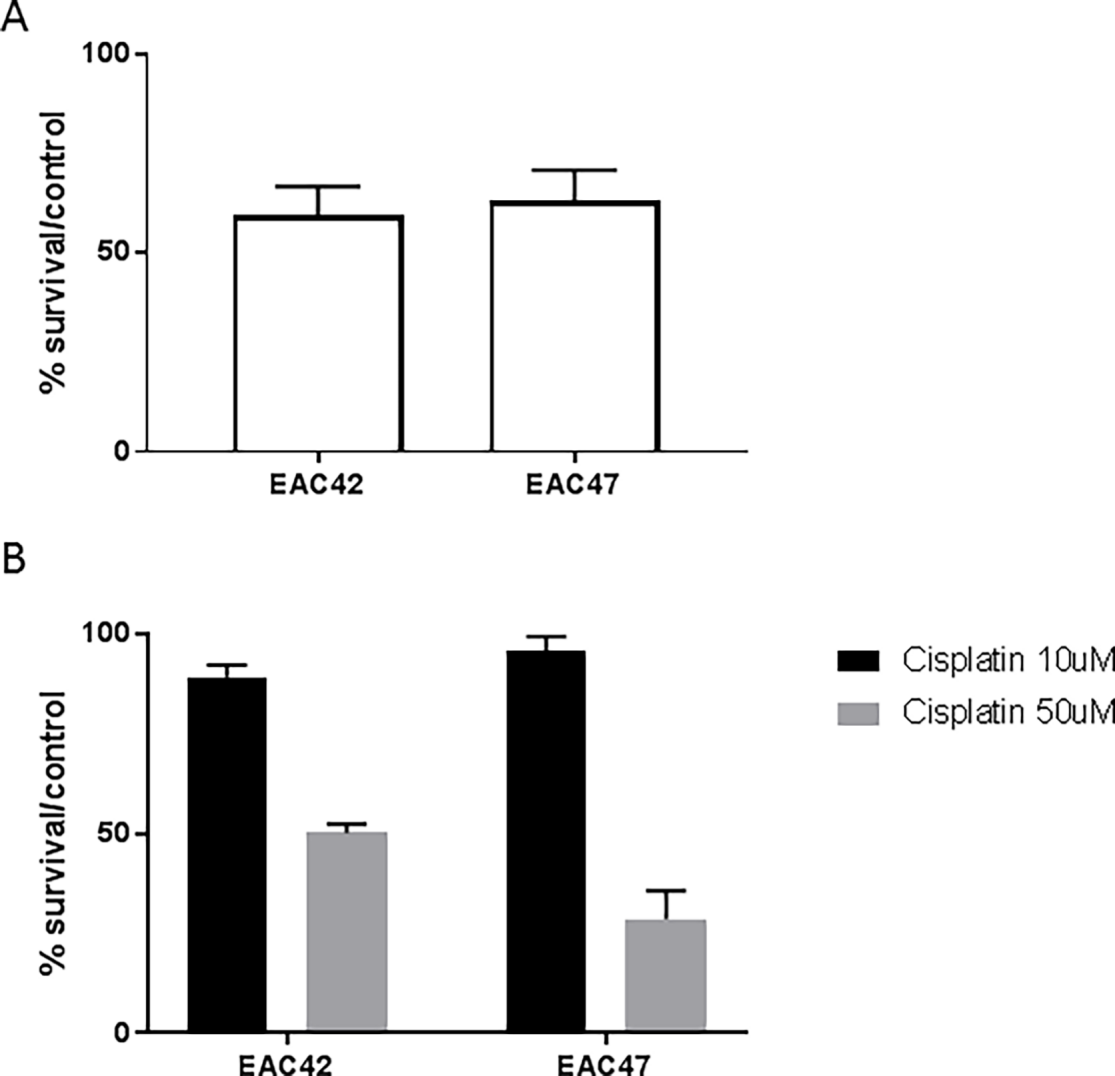
Clonogenic survival assay. Clonogenic survival in response to treatment with (A) 10nM gemcitabine and (B) 10μM or 50μM of cisplatin. Error bars represent SD.

Gemcitabine (Gemzar) was the 3^rd^ and 6^th^ treatment suggestion in EAC42 and EAC47, respectively (Fig 3). Cisplatin on the other hand did not result in a reduction of cell survival in either patient-derived line at a concentration of 10μM. In order to verify the results of the DST screen, we performed clonogenic survival assays using EAC42 and EAC47. The calculated EC_50_ for gemcitabine of 6.6nM for EAC42 and 10.7nM for EAC47. No EC_50_ was determined for cisplatin treatment. Treatment with 10nM of gemcitabine for 2h reduced survival to 59.26% in EAC42 and 63.28% in EAC47 (Fig 4A). Treatment with 10μM and 50μM of cisplatin for 24h reduced survival to 89.19% and 50.1%, respectively in EAC42. Treatment of EAC47 reduced survival to 95.75% in response to treatment with 10μM cisplatin and 28.64% in response to treatment with 50μM cisplatin (Fig 4B).

### Epigenetic compound library screen

OE33, Flo-1 and EAC47 cells were exposed to a 163 compound library containing epigenetic modifiers at a concentration of 6μM. Positive hits were defined as any compound that showed cell killing higher that 3 standard deviations of the negative control.

**Fig 5:**
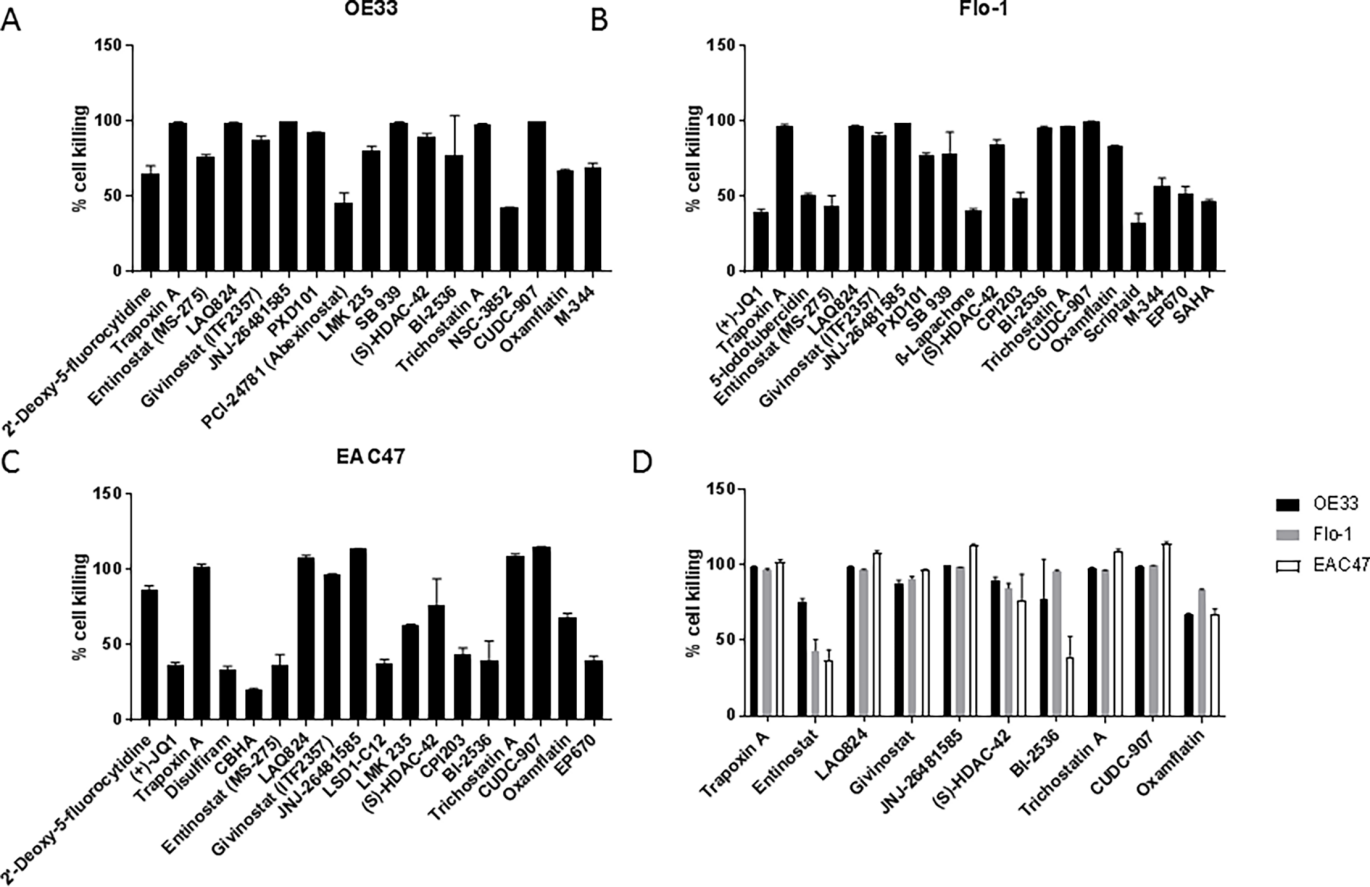
Epigenetics compound library screen. Percent cell killing in response to treatment with the epigenetics compound library in (A) OE33, (B) Flo-1 and (C) EAC47. (D) 10 compounds displayed significant reductions of cell viability in all of the tested cell lines.

17 compounds displayed a significant reduction in cell viability in OE33, the majority of which were HDAC inhibitors (Fig 5A). Flo-1 and EAC47 cell viability was reduced by 20 compounds (Fig 5B) and 18 compounds (Fig 5C), respectively. In addition to HDAC inhibitors, Flo-1 and EAC47 display sensitivity towards the BET-inhibitors JQ1 and CPI203 (S3 Table).

10 compounds displayed a significant reduction of cell survival in all of the tested cell lines (Fig 5D). With the exception of the PLK inhibitor Bl-2536, all common hits are HDAC inhibitors.

## Discussion

Treatment stratification of patients with esophageal adenocarcinoma is problematic due to the lack of molecular markers predicting outcome and treatment response, and the optimal patient management remains controversial. While the collection of large sequencing data sets revealed genetic changes, including alterations in DNA methylation patterns [4, 6–8], these datasets can only supplement the histological classification without providing patient-specific treatment suggestions. Additionally, the aggressive chemotherapy regimen commonly used in esophageal cancer patients come at the price of high normal tissue toxicity. This is especially critical in the light of the high rate of unsuccessful treatment attempts observed in this patient population. *Ex vivo* DST screening may present a new stratification approach for patients with esophageal adenocarcinoma that allows the identification of patient-specific treatments while reducing normal tissue toxicity [14].

The esophageal adenocarcinoma cell lines display a wide range of treatment responses and vastly different individual sensitivity profiles. In contrast to our previous observations in AML, we uncovered a small subset of compounds that displayed treatment efficacy in all of the tested samples. Specifically, Bortezomib displayed high activity in all lines. The DST screen revealed a number of treatment options not part of standard therapy in esophageal adenocarcinoma, while compounds commonly used in patients such as platinum drugs (cisplatin, carboplatin, oxaliplatin), taxanes (docetaxel, placlitaxel), 5-FU, irinotecan, capecitabine and epirubicin display treatment efficacy in few or none of the lines. However, similar to previous observations, a treatment response can be observed in response to treatment with cisplatin when doses are escalated beyond the maximum dose of the DST screen. Due to high levels of normal tissue toxicity and numerous difficult to manage side effects, a similar dose escalation is not feasible in a clinical setting. The dose range used on the DST screen was chosen to represent a clinically relevant treatment dose. Therefore, a negative result on the DST screen does not imply a complete lack of treatment response. Nevertheless, as seen in the case of cisplatin treatment, the dose needed for a clinically relevant response lies outside of the dose range that is clinically feasible. The data derived from the FDA screen suggests that phenotypic-based treatment stratification may present a way to overcome the current issues with patient stratification and classification.

The impact of epigenetic modifications has recently come into the focus of the esophageal adenocarcinoma community and studies have shown numerous changes in epigenetic marks in patient samples. A small number of studies have provided encouraging results for the use of HDAC inhibitors for the treatment of esophageal cancer [17–20]. We observed high levels of treatment responses towards a panel of HDAC inhibitors using an epigenetic library screen containing 163 compounds targeting epigenetic modifiers. This suggests that compounds targeting epigenetic modifiers in esophageal adenocarcinoma should be added to the screening library and may present a promising therapeutic option of sensitive patients.

A pilot trial evaluating a set of patients with esophageal adenocarcinoma will be necessary to examine the clinical feasibility of this precision medicine approach. Clinically the treatment of esophageal adenocarcinoma consist of a multimodal approach. Although our platform evaluates the response towards single treatments only, our experience using the DST platform for the treatment stratification of patients with relapsed/refractory AML [14] suggests that rational combinations can be successfully added to the DST suggestion.

Although all patients receive at least one biopsy, the material obtained during the procedure is essential for diagnostic purposes and the material superfluous to pathological staging is rarely of sufficient quantity and quality to support and *ex vivo* screen of this magnitude. This can be overcome by the use of surgical specimen which yield sufficient cellular material to support the DST screen. Although a matched normal tissue sample represents the ideal toxicity control for the DST screen, normal esophageal epithelium is not obtainable from either biopsies or surgical samples. White blood cells, while a matched control, display a vastly different sensitivity profile than the esophageal epithelium and cannot be used to evaluate epithelial toxicity in the esophagus. This can be overcome by using a catchment area matched panel of normal esophageal epithelium samples as a normal reference for future patient screens.

This precision medicine approach has the potential to improve treatment stratification and outcome for patients with esophageal adenocarcinoma. Additionally, DST screens on patient populations may allow us further insight into the biology of the disease through the accumulation of sensitivity information on different classes of therapeutic compounds.

## Supporting information

S1 Fig Individual screening results. Bar graphs of clinically actionable drug responses for (A) Flo1 and (B) SK-GT2.
(TIF)

S1 Table DSS and EC_50_ values for normal esophageal cells. (DOCX)

S2 Table sDSS_mod_ values. (DOCX)

S3 Table Results of the epigenetic modifier screen. (DOCX)
