# Supplementary figures and images for: Ex vivo drug sensitivity testing as a means for drug repurposing in esophageal adenocarcinoma
Source: PLoS One. 2018 Sep 13;13(9):e0203173. doi: 10.1371/journal.pone.0203173 (PMC6136712; doi:10.1371/journal.pone.0203173)

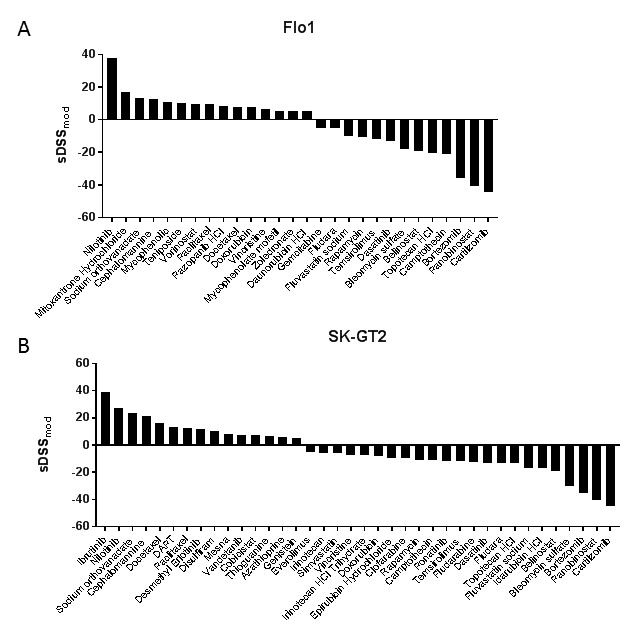

Supplement: S1 Fig — Bar graphs of clinically actionable drug responses for (A) Flo1 and (B) SK-GT2. (TIF) [file pone.0203173.s001.tif]
