# Supplementary material for: Ex vivo drug sensitivity testing as a means for drug repurposing in esophageal adenocarcinoma
Source: PLoS One. 2018 Sep 13;13(9):e0203173. doi: 10.1371/journal.pone.0203173 (PMC6136712; doi:10.1371/journal.pone.0203173)
Supplement: S1 Table — (DOCX) [file pone.0203173.s002.docx]

| **Compound name** | **EC_50_ (uM)** | **DSS** |
| --- | --- | --- |
| 10-DAB | 5.48 | 0.01 |
| 2-Methoxyestradiol | 35.52 | 0.26 |
| Abiraterone | N/A | 0 |
| Abiraterone Acetate | N/A | 0 |
| Abitrexate | 0.04 | 21.14 |
| Adenine | N/A | 0 |
| Adenine hydrochloride | 0.01 | 0 |
| Adenine sulfate | 0.25 | 0 |
| Adrucil | 0.92 | 4.47 |
| Afatinib | 4.61 | 0.12 |
| Altretamine | N/A | 0 |
| Aminoglutethimide | N/A | 0 |
| Anagrelide HCl | 0.04 | 0 |
| Anastrozole | 1.59 | 0 |
| Apatinib | 0.05 | 0 |
| Aprepitant | N/A | 0 |
| Artemether | 2.90E-07 | 0 |
| Aspirin | 2.50E+62 | 0 |
| Atazanavir sulfate | N/A | 0 |
| Axitinib | 6.26E+22 | 0 |
| Azacitidine | N/A | 0 |
| Azaguanine-8 | 3312.9 | 2.04 |
| Azathioprine | 1.94E+12 | 0.02 |
| Azithromycin | N/A | 0 |
| Bacitracin zinc | N/A | 0 |
| Belinostat | 1.08 | 19 |
| Bendamustine HCL | N/A | 0 |
| Bepotastine Besilate | N/A | 0 |
| Bergapten | 0.05 | 0 |
| Betapar | N/A | 0 |
| Bexarotene | 4.29 | 0.12 |
| Bicalutamide | N/A | 0 |
| Bindarit | 0.002 | 0.03 |
| Bleomycin sulfate | 0.37 | 30.58 |
| Blonanserin | 0.04 | 0 |
| Bortezomib | 0.2 | 35.58 |
| Bosutinib | 3.39E+14 | 0 |
| Busulfan | N/A | 0 |
| Cabozantinib | 3.00E+172 | 0 |
| Camptothecin | 0.02 | 52.69 |
| Capecitabine | 1 | 0 |
| Carbazochrome sodium sulfonate | N/A | 0 |
| Carboplatin | 2.37 | 3.68 |
| Carmofur | 0.71 | 4.76 |
| Cartilzomib | 0.08 | 44.43 |
| Celecoxib | N/A | 0 |
| Cephalomannine | 0.161532 | 23.35 |
| Cisplatin | 0.01 | 0 |
| Cladribine | 2.02E+08 | 0.13 |
| Clofarabine | 0.07 | 19.89 |
| Clomifene citrate | N/A | 0 |
| Clorsulon | N/A | 0 |
| Cobicistat | 6.06E+11 | 0 |
| Cortisone acetate | N/A | 0 |
| Crizotinib | 8.03 | 2.75 |
| Cyclophosphamide monohydrate | N/A | 0 |
| Cytarabine | 11.79879 | 1.34 |
| Dacarbazine | N/A | 0 |
| DAPT | 6.42E+33 | 0 |
| Dasatinib | 0.08 | 12.81 |
| Daunorubicin HCl | 0.98 | 21.12 |
| Decitabine | 0.17 | 0 |
| Desmethyl Erlotinib | 3687.11 | 3.34 |
| Dexamethasone | N/A | 0 |
| Dexamethasone acetate | N/A | 0 |
| Diethylstilbestrol | 0.01 | 0 |
| Disulfiram | N/A | 0 |
| Docetaxel | 3 | 40.14 |
| Dorzolamide HCL | 5.33 | 0.01 |
| Doxercalciferol | 0.007 | 0 |
| Doxorubicin | 1.42 | 15.67 |
| Ellagic acid | 3.20E+117 | 0 |
| Eltrombopag | 4.66458 | 0.32 |
| Enzalutamide | 3.03E+08 | 0 |
| Epinephrine bitartrate | N/A | 0 |
| Epirubicin Hydrochloride | 1.2 | 18.98 |
| Erlotinib HCl | 3.13E+46 | 0 |
| Esomeprazole sodium | N/A | 0 |
| Estradiol | N/A | 0 |
| Estrone | N/A | 0 |
| Etoposide | 11.74 | 4.68 |
| Everolimus | 9 | 7.57 |
| Evista | N/A | 0 |
| Exemestane | N/A | 0 |
| Ezetimibe | N/A | 0 |
| Famciclovir | 1.12 | 0 |
| Febuxostat | 6335.33 | 0 |
| Floxuridine | 0.83 | 4.9 |
| Fludara | 0.05 | 46.34 |
| Fludarabine | 0.07 | 42.19 |
| Flunarizine 2HCl | 2.56E-10 | 0.03 |
| Flutamide | 5.24E+41 | 0 |
| Fluvastatin sodium | 1.06 | 17.36 |
| Ftorafur | N/A | 0 |
| Fulvestrant | 6.48E+33 | 0 |
| Gadodiamide | 9.88E+38 | 0 |
| Gefitinib | 1.55E+31 | 0 |
| Gemcitabine | 0.32 | 17.72 |
| Gemzar | 0.77 | 16.2 |
| Geniposidic acid | N/A | 0 |
| Genistein | 19.44 | 0.04 |
| Hydrocortisone | N/A | 0 |
| Hydroxyurea | 1.70E+40 | 0 |
| Hygromycin B | 7.77 | 3.8 |
| Ibrutinib | N/A | 0 |
| Idarubicin HCl | 0.71 | 26.1 |
| Ifosfamide | N/A | 0 |
| Imatinib | 2.91E+34 | 0 |
| Imatinib Mesylate | 3.08E+11 | 0 |
| Imiquimod | 2 | 0 |
| Irinotecan | 5.11 | 6.99 |
| Irinotecan HCl Trihydrate | 1.57 | 13.38 |
| Isotretinoin | N/A | 0 |
| Itraconazole | N/A | 0 |
| Lamotrigine | 3.08E+37 | 0 |
| Lapatinib | 0.04 | 0 |
| Lapatinib Ditosylate | N/A | 0 |
| L-Arginine HCl | 0.01 | 0 |
| Lenalidomide | 1.14E+76 | 0 |
| Letrozole | 0.06 | 0 |
| Leucovorin Calcium | 0.09 | 0.04 |
| Linagliptin | 0.38 | 0 |
| Lincomycin hydrochloride | 0.99 | 0 |
| Lomustine | N/A | 0 |
| Lonidamine | N/A | 0 |
| Maraviroc | N/A | 0 |
| Masitinib | N/A | 0 |
| Medroxyprogesterone acetate | N/A | 0 |
| Megestrol Acetate | N/A | 0 |
| Mercaptopurine | 103.98 | 0.05 |
| Mesna | N/A | 0 |
| Methacycline hydrochloride | 18.81 | 0 |
| Methazolastone | N/A | 0 |
| Mifepristone | N/A | 0 |
| Mirabegron | 0.02 | 0 |
| Mitotane | 0.006 | 0 |
| Mitoxantrone Hydrochloride | 1.67 | 17.16 |
| Mizoribine | 0.006 | 0 |
| MLN-4924 | 21949.02 | 0.51 |
| Moroxydine |  | 0 |
| Mycophenolate mofetil | 2.18 | 2.66 |
| Mycophenolic | 15.67 | 2.17 |
| Naloxone HCl | 0.15 | 0 |
| Nelarabine | N/A | 0 |
| Nilotinib | 14.75913 | 0.43 |
| Nilvadipine | 0.01 | 0 |
| Noscapine HCl | N/A | 0 |
| Ofloxacin | N/A | 0 |
| Oxaliplatin | 21.99 | 0.06 |
| Paclitaxel | 0.01 | 34.94 |
| Paeoniflorin | N/A | 0 |
| Pamidronate Disodium | 24.97 | 0 |
| Panobinostat | 0.09 | 40.42 |
| Pazopanib | 7.57E+12 | 0 |
| Pazopanib HCl | 509.08 | 0.07 |
| Pemetrexed | 0.06 | 16.15 |
| Phenylbutazone | N/A | 0 |
| Pimecrolimus | 5.3 | 1.11 |
| Pioglitazone | N/A | 0 |
| Pomalidomide | 0.000158 | 0 |
| Ponatinib | 2.81 | 11.77 |
| Prednisone | N/A | 0 |
| Procarbazine HCl | N/A | 0 |
| Ranolazine | N/A | 0 |
| Rapamycin | 3 | 11.01 |
| Regorafenib | 25.62 | 1.85 |
| Rosiglitazone | 4.53E+55 | 0 |
| Ruxolitinib | N/A | 0 |
| Simvastatin | 3.35 | 5.66 |
| Sodium butyrate | N/A | 0 |
| Sodium orthovanadate | N/A | 0 |
| Sorafenib | 9.62 | 1.79 |
| Streptozotocin | 5.58E+51 | 0 |
| Sulindac | 0.05 | 0 |
| Sunitinib Malate | 0.01 | 0 |
| Tacrolimus | N/A | 0 |
| TAME | N/A | 0 |
| Tamoxifen Citrate | N/A | 0 |
| Temocapril HCl | N/A | 0 |
| Temsirolimus | 12.92 | 11.93 |
| Teniposide | 5.17 | 6.79 |
| Thalidomide | N/A | 0 |
| Thioguanine | 3.16 | 4.62 |
| Tofacitinib citrate | 2.10E+10 | 0 |
| Tolbutamide | 0.06 | 0 |
| Tolnaftate | 1.75E+86 | 0 |
| Topotecan HCl | 0.06 | 43.21 |
| Toremifene Citrate | N/A | 0 |
| Tretinoin | N/A | 0 |
| Triamcinolone Acetonide | N/A | 0 |
| Ubenimex | 7.66E+08 | 0 |
| Valproic acid sodium salt | 2 | 0 |
| Vandetanib | 4.46 | 0.13 |
| Vemurafenib | 0.05 | 0 |
| Vinblastine | 529.58 | 1.63 |
| Vincristine | 0.03 | 26.9 |
| Vinpocetine | N/A | 0 |
| Vismodegib | N/A | 0 |
| Vorinostat | 4.41 | 5.09 |
| Zileuton | N/A | 0 |
| Zoledronic Acid | N/A | 0 |
