# Supplementary material for: Ex vivo drug sensitivity testing as a means for drug repurposing in esophageal adenocarcinoma
Source: PLoS One. 2018 Sep 13;13(9):e0203173. doi: 10.1371/journal.pone.0203173 (PMC6136712; doi:10.1371/journal.pone.0203173)
Supplement: S2 Table — (DOCX) [file pone.0203173.s003.docx]

|  | **compound** | **sDSS_mod_** |  | **compound** | **sDSS_mod_** |  |
| --- | --- | --- | --- | --- | --- | --- |
| **EAC42** | Floxuridine | 40.60868 | **EAC47** | Abitrexate | 35.78047 |  |
|  | Abitrexate | 31.2825 |  | Floxuridine | 35.53781 |  |
|  | Gemzar | 17.37189 |  | Pemetrexed | 14.75255 |  |
|  | MLN-4924 | 17.05494 |  | Mercaptopurine | 10.90497 |  |
|  | Gemcitabine | 14.66229 |  | MLN-4924 | 10.87256 |  |
|  | Pemetrexed | 14.61893 |  | Gemzar | 10.02646 |  |
|  | Belinostat | 10.90404 |  | Gemcitabine | 8.406872 |  |
|  | Mercaptopurine | 10.48278 |  | Thioguanine | 8.363869 |  |
|  | Thioguanine | 9.961284 |  | Azathioprine | 7.782749 |  |
|  | Zoledronate | 9.832542 |  | Belinostat | 6.845786 |  |
|  | Mycophenolic | 8.479274 |  | Zoledronate | 6.525879 |  |
|  | Cladribine | 7.958989 |  | Mycophenolic | 5.9818 |  |
|  | Panobinostat | 7.607243 |  | Cladribine | 5.947152 |  |
|  | Vorinostat | 7.485339 |  | Vorinostat | 5.288429 |  |
|  | Disulfiram | 7.111539 |  | Mitoxantrone Hydrochloride | 5.241739 |  |
|  | Mitoxantrone Hydrochloride | 6.699238 |  | Irinotecan | -5.5855 |  |
|  | Idarubicin HCl | 6.506181 |  | Simvastatin | -5.65964 |  |
|  | Mycophenolate mofetil | 6.416769 |  | Everolimus | -5.69691 |  |
|  | Azathioprine | 6.181488 |  | Irinotecan HCl Trihydrate | -6.3823 |  |
|  | Azaguanine-8 | 6.076946 |  | Cephalomannine | -8.86771 |  |
|  | Etoposide | 5.048554 |  | Rapamycin | -8.93077 |  |
|  | Temsirolimus | -5.1083 |  | Temsirolimus | -9.99363 |  |
|  | Simvastatin | -5.36269 |  | Clofarabine | -10.1317 |  |
|  | Cephalomannine | -5.55249 |  | Dasatinib | -10.2728 |  |
|  | Rapamycin | -6.15001 |  | Topotecan HCl | -11.6273 |  |
|  | Irinotecan HCl Trihydrate | -6.56306 |  | Ponatinib | -11.7731 |  |
|  | Vincristine | -7.91997 |  | Fluvastatin sodium | -15.1417 |  |
|  | Clofarabine | -8.02444 |  | Camptothecin | -15.9763 |  |
|  | Topotecan HCl | -9.36679 |  | Vincristine | -16.3015 |  |
|  | Dasatinib | -9.75152 |  | Paclitaxel | -17.6632 |  |
|  | Ponatinib | -11.7731 |  | Docetaxel | -23.2183 |  |
|  | Paclitaxel | -12.1 |  | Bortezomib | -23.5292 |  |
|  | Camptothecin | -13.5945 |  | Bleomycin sulfate | -29.4923 |  |
|  | Fluvastatin sodium | -14.3727 |  | Fludarabine | -42.19 |  |
|  | Docetaxel | -15.5932 |  | Fludara | -46.3431 |  |
|  | Bortezomib | -21.0264 | **OE33** | Thioguanine | 42.57923 |  |
|  | Bleomycin sulfate | -29.1318 |  | Teniposide | 39.44608 |  |
|  | Fludarabine | -42.0707 |  | Nilotinib | 32.09245 |  |
|  | Fludara | -46.3431 |  | Gadodiamide | 29.61532 |  |
| **OE19** | Ibrutinib | 31.18079 |  | Simvastatin | 26.14689 |  |
|  | Nilotinib | 18.74106 |  | Ranolazine | 25.88701 |  |
|  | Vorinostat | 9.843322 |  | Pemetrexed | 25.31537 |  |
|  | Vandetanib | 7.027033 |  | Azacitidine | 23.96342 |  |
|  | Sodium orthovanadate | 5.913396 |  | Toremifene Citrate | 23.6335 |  |
|  | Simvastatin | -5.64699 |  | Crizotinib | 22.73883 |  |
|  | Irinotecan | -6.33963 |  | Cabozantinib | 19.54812 |  |
|  | Rapamycin | -11.012 |  | Bacitracin zinc | 17.06978 |  |
|  | Epirubicin Hydrochloride | -11.4055 |  | Abitrexate | 16.68859 |  |
|  | Ponatinib | -11.756 |  | Mycophenolic | 16.22515 |  |
|  | Temsirolimus | -11.9329 |  | Clofarabine | 16.0076 |  |
|  | Gemzar | -11.949 |  | Vorinostat | 15.70575 |  |
|  | Irinotecan HCl Trihydrate | -12.0524 |  | Idarubicin HCl | 15.05771 |  |
|  | Dasatinib | -12.8072 |  | Floxuridine | 13.66626 |  |
|  | Doxorubicin | -12.9053 |  | Carmofur | 12.41689 |  |
|  | Daunorubicin HCl | -13.6679 |  | Irinotecan HCl Trihydrate | 12.06526 |  |
|  | Pemetrexed | -15.3102 |  | Gemzar | 11.78554 |  |
|  | Fluvastatin sodium | -16.7184 |  | Pazopanib HCl | 11.37017 |  |
|  | Gemcitabine | -16.819 |  | Mycophenolate mofetil | 11.02724 |  |
|  | Bleomycin sulfate | -18.9933 |  | Adrucil | 10.55561 |  |
|  | Belinostat | -19.002 |  | Gemcitabine | 9.442599 |  |
|  | Clofarabine | -19.554 |  | Mitoxantrone Hydrochloride | 9.033405 |  |
|  | Fludarabine | -20.7262 |  | Desmethyl Erlotinib | 8.647554 |  |
|  | Abitrexate | -21.1435 |  | Azaguanine-8 | 8.321793 |  |
|  | Idarubicin HCl | -22.4296 |  | Vandetanib | 8.254553 |  |
|  | Fludara | -23.9486 |  | Paclitaxel | 7.641975 |  |
|  | Camptothecin | -34.9341 |  | Ellagic acid | 7.542475 |  |
|  | Bortezomib | -35.5757 |  | Epirubicin Hydrochloride | 6.778189 |  |
|  | Topotecan HCl | -36.6414 |  | Etoposide | 6.550301 |  |
|  | Panobinostat | -40.4215 |  | Cytarabine | 6.525241 |  |
|  | Cartilzomib | -42.8127 |  | Cephalomannine | 5.452001 |  |
| **Flo-1** | Nilotinib | 37.79776 |  | Irinotecan | 5.245792 |  |
|  | Mitoxantrone Hydrochloride | 17.0838 |  | Genistein | 5.078667 |  |
|  | Sodium orthovanadate | 13.33495 |  | Everolimus | -5.49266 |  |
|  | Cephalomannine | 12.79157 |  | Ponatinib | -6.15505 |  |
|  | Mycophenolic | 10.91715 |  | Vincristine | -8.23422 |  |
|  | Teniposide | 10.22 |  | Fludarabine | -9.24847 |  |
|  | Vorinostat | 9.58 |  | Fluvastatin sodium | -10.5948 |  |
|  | Paclitaxel | 9.33 |  | Rapamycin | -11.012 |  |
|  | Pazopanib HCl | 8.16 |  | Temsirolimus | -11.9329 |  |
|  | Docetaxel | 8.04 |  | Dasatinib | -12.8072 |  |
|  | Doxorubicin | 7.66 |  | Fludara | -12.9868 |  |
|  | Vincristine | 6.31 |  | Bleomycin sulfate | -15.1946 |  |
|  | Mycophenolate mofetil | 5.44 |  | Belinostat | -19.002 |  |
|  | Zoledronate | 5.26 |  | Bortezomib | -35.5757 |  |
|  | Daunorubicin HCl | 5.15 |  | Panobinostat | -40.4215 |  |
|  | Gemcitabine | -5 |  | Cartilzomib | -43.92 |  |
|  | Fludara | -5.23 | **SK-GT2** | Ibrutinib | 39.30835 |  |
|  | Fluvastatin sodium | -9.71 |  | Nilotinib | 27.22125 |  |
|  | Rapamycin | -10.72 |  | Sodium orthovanadate | 23.65748 |  |
|  | Temsirolimus | -11.93 |  | Cephalomannine | 21.46337 |  |
|  | Dasatinib | -12.8 |  | Docetaxel | 16.5247 |  |
|  | Bleomycin sulfate | -18.12 |  | DAPT | 13.53776 |  |
|  | Belinostat | -19 |  | Paclitaxel | 12.80242 |  |
|  | Topotecan HCl | -20.26 |  | Desmethyl Erlotinib | 11.77284 |  |
|  | Camptothecin | -20.76 |  | Disulfiram | 10.36181 |  |
|  | Bortezomib | -35.57 |  | Mesna | 8.020772 |  |
|  | Panobinostat | -40.42 |  | Vandetanib | 7.721034 |  |
|  | Cartilzomib | -44.35 |  | Cobicistat | 7.684683 |  |
|  |  |  |  | Thioguanine | 6.83846 |  |
|  |  |  |  | Azathioprine | 5.73305 |  |
|  |  |  |  | Genistein | 5.116558 |  |
|  |  |  |  | Everolimus | -5.05368 |  |
|  |  |  |  | Irinotecan | -5.57392 |  |
|  |  |  |  | Simvastatin | -5.65964 |  |
|  |  |  |  | Vincristine | -7.21044 |  |
|  |  |  |  | Irinotecan HCl Trihydrate | -7.5553 |  |
|  |  |  |  | Doxorubicin | -8.36482 |  |
|  |  |  |  | Epirubicin Hydrochloride | -9.53458 |  |
|  |  |  |  | Clofarabine | -9.55873 |  |
|  |  |  |  | Rapamycin | -11.012 |  |
|  |  |  |  | Camptothecin | -11.3049 |  |
|  |  |  |  | Ponatinib | -11.7711 |  |
|  |  |  |  | Temsirolimus | -11.9329 |  |
|  |  |  |  | Fludarabine | -12.4173 |  |
|  |  |  |  | Dasatinib | -12.8072 |  |
|  |  |  |  | Fludara | -12.9068 |  |
|  |  |  |  | Topotecan HCl | -13.0332 |  |
|  |  |  |  | Fluvastatin sodium | -17.1525 |  |
|  |  |  |  | Idarubicin HCl | -17.1644 |  |
|  |  |  |  | Belinostat | -19.002 |  |
|  |  |  |  | Bleomycin sulfate | -29.9671 |  |
|  |  |  |  | Bortezomib | -35.5649 |  |
|  |  |  |  | Panobinostat | -40.4215 |  |
|  |  |  |  | Cartilzomib | -44.432 |  |
