# Supplementary material for: Ex vivo drug sensitivity testing as a means for drug repurposing in esophageal adenocarcinoma
Source: PLoS One. 2018 Sep 13;13(9):e0203173. doi: 10.1371/journal.pone.0203173 (PMC6136712; doi:10.1371/journal.pone.0203173)
Supplement: S3 Table — (DOCX) [file pone.0203173.s004.docx]

|  | **Compound** | **% cell killing /control** | **SD** |
| --- | --- | --- | --- |
| **OE33** | 2'-Deoxy-5-fluorocytidine | 65.19 | 5.15 |
|  | Trapoxin A | 98.89 | 0.40 |
|  | Entinostat (MS-275) | 75.99 | 1.68 |
|  | LAQ824 | 98.97 | 0.27 |
|  | Givinostat (ITF2357) | 87.68 | 2.24 |
|  | JNJ-26481585 | 99.54 | 0.01 |
|  | PXD101 | 92.27 | 0.54 |
|  | PCI-24781 (Abexinostat) | 45.97 | 6.20 |
|  | LMK 235 | 80.31 | 3.11 |
|  | SB 939 | 98.89 | 0.40 |
|  | (S)-HDAC-42 | 89.27 | 2.50 |
|  | BI-2536 | 77.59 | 26.15 |
|  | Trichostatin A | 98.02 | 0.37 |
|  | NSC-3852 | 42.71 | 0.21 |
|  | CUDC-907 | 99.37 | 0.03 |
|  | Oxamflatin | 67.45 | 0.43 |
|  | M-344 | 68.75 | 3.43 |
| **Flo-1** | (+)-JQ1 | 39.15 | 2.26 |
|  | Trapoxin A | 96.37 | 1.48 |
|  | 5-Iodotubercidin | 50.40 | 1.49 |
|  | Entinostat (MS-275) | 43.31 | 7.21 |
|  | LAQ824 | 96.83 | 0.25 |
|  | Givinostat (ITF2357) | 90.88 | 1.46 |
|  | JNJ-26481585 | 98.39 | 0.04 |
|  | PXD101 | 77.56 | 1.22 |
|  | SB 939 | 78.84 | 13.87 |
|  | β-Lapachone | 40.89 | 1.09 |
|  | (S)-HDAC-42 | 84.63 | 3.06 |
|  | CPI203 | 48.79 | 3.98 |
|  | BI-2536 | 95.90 | 0.79 |
|  | Trichostatin A | 96.32 | 0.25 |
|  | CUDC-907 | 99.74 | 0.13 |
|  | Oxamflatin | 83.66 | 0.25 |
|  | Scriptaid | 32.49 | 6.21 |
|  | M-344 | 56.82 | 5.35 |
|  | SAHA | 46.32 | 1.47 |
| **EAC47** | 2'-Deoxy-5-fluorocytidine | 86.83 | 2.41 |
|  | (+)-JQ1 | 36.01 | 2.33 |
|  | Trapoxin A | 102.19 | 1.28 |
|  | Disulfiram | 33.87 | 1.86 |
|  | CBHA | 20.30 | 0.72 |
|  | Entinostat (MS-275) | 36.56 | 7.18 |
|  | LAQ824 | 108.01 | 1.34 |
|  | Givinostat (ITF2357) | 96.79 | 0.38 |
|  | JNJ-26481585 | 113.73 | 0.17 |
|  | LSD1-C12 | 37.25 | 2.98 |
|  | LMK 235 | 62.72 | 1.09 |
|  | (S)-HDAC-42 | 76.52 | 17.28 |
|  | CPI203 | 43.29 | 4.70 |
|  | BI-2536 | 39.52 | 13.06 |
|  | Trichostatin A | 109.49 | 0.99 |
|  | CUDC-907 | 114.62 | 0.69 |
|  | Oxamflatin | 67.71 | 3.26 |
